# Vitamin D for Recovery of COVID-19 in Patients With Chronic Kidney Disease

**DOI:** 10.3389/fnut.2022.930176

**Published:** 2022-06-15

**Authors:** Wen-Fang Chiang, Po-Jen Hsiao, Jenq-Shyong Chan

**Affiliations:** ^1^Division of Nephrology, Department of Medicine, Armed Forces Taoyuan General Hospital, Taoyuan, Taiwan; ^2^Division of Nephrology, Department of Medicine, Tri-Service General Hospital, National Defense Medical Center, Taipei, Taiwan; ^3^School of Medicine, National Defense Medical Center, Taipei, Taiwan

**Keywords:** chronic kidney disease, COVID-19, immune system, treatment, vitamin D

## Abstract

The severity of coronavirus disease 2019 (COVID-19) is determined not only by viral damage to cells but also by the immune reaction in the host. In addition to therapeutic interventions that target the viral infection, immunoregulation may be helpful in the management of COVID-19. Vitamin D exerts effects on both innate and adaptive immunity and subsequently modulates immune responses to bacteria and viruses. Patients with chronic kidney disease (CKD) frequently have vitamin D deficiency and increased susceptibility to infection, suggesting a potential role of vitamin D in this vulnerable population. In this paper, we review the alterations of the immune system, the risk of COVID-19 infections and mechanisms of vitamin D action in the pathogenesis of COVID-19 in CKD patients. Previous studies have shown that vitamin D deficiency can affect the outcomes of COVID-19. Supplementing vitamin D during treatment may be protective against COVID-19. Future studies, including randomized control trials, are warranted to determine the effect of vitamin D supplementation on the recovery from COVID-19 in CKD patients.

## Introduction

The emergence of severe acute respiratory syndrome coronavirus 2 (SARS-CoV-2) caused the major devastating coronavirus disease 2019 (COVID-19) pandemic. Although the symptoms of most infected patients are mild to moderate, some may quickly progress to a life-threatening condition (1). SARS-CoV-2 can cause lung tissue damage, resulting in acute respiratory distress syndrome (ARDS), which is usually accompanied by sepsis and septic shock, both of which are leading causes of mortality (2). ARDS is a consequence of uncontrolled inflammatory cytokine production and oxidative stress in the lungs following viral infection (3). Chronic kidney disease (CKD) is characterized by the retention of uraemic toxins and cytokines, leading to the coexistence of immunoactivation and immunodepression, and is a major risk factor for poor prognosis of COVID-19. Therefore, it is crucial to discern the immunopathologic process underlying SARS-CoV-2 infection to identify appropriate management strategies for COVID-19 in this vulnerable population.

Although specific antiviral agents against SARS-CoV-2 have been approved by the US Food and Drug Administration, an effective cure remains lacking. Thus, there is an urgent need to seek alternative and timely treatments for the disease. Since patients with CKD usually have suboptimal nutrient intake and chronic systemic inflammation, supplementing certain nutrients may be helpful in recovery from COVID-19. Vitamin D, a widely available, inexpensive and harmless supplement, may potentially have a significant effect on reducing COVID-19 severity in CKD patients. This review was designed to discuss the pathophysiology that may occur in CKD patients with COVID-19 and how vitamin D administration can contribute to modulating the immune system and alleviating the pathological consequences of COVID-19.

## Alterations of the Immune System in CKD Patients

### Normal Immune Reaction

There are two major subsystems of the immune system, the innate and adaptive immune systems. The innate immune system is a primary defense mechanism against invading organisms and consists of cellular and humoral defenses against pathogens. The cellular components involve a variety of different types of leukocytes, including monocytes, macrophages, neutrophils, dendritic cells, endothelial cells, and humoral components such as C-reactive protein, lysozymes, and complement. Innate immune cells can recognize invading microorganisms via pathogen-recognition receptors (PRRs), namely, Toll-like receptors (TLRs), which then stimulate immune cells to release cytokines and various antimicrobial peptides (AMPs) (4, 5). The adaptive immune system acts as a second line of host defense and comprises the immune response through the activation of antigen-presenting cells (APCs), such as dendritic cells, and the antigen recognition cells, T and B lymphocytes. Adaptive immune cells learn to recognize foreign molecules the first time they are encountered, retain their memory and identify these molecules in subsequent encounters. APCs use major histocompatibility complex (MHC) molecules to present antigens and interact with naïve T cells that are converted to activated effector T cells. There are three subsets of effector T cells: cytotoxic T cells and T helper 1 and 2 cells. T helper 2 cells can stimulate B cells to produce antibodies.

### CKD and Immune Dysfunction

In addition to removal of metabolic waste materials and medicines from the body, the kidneys play an important role in the clearance of circulating cytokines and bacterial toxins and in the continuous sampling of blood-borne proteins, contributing to homeostasis of the immune system. A decline in renal function that persists for >3 months is referred to as CKD and is associated with profound alterations in immune function, including immune activation, marked by systemic inflammation and acquired immunosuppression (6). Systemic inflammation leads to atherosclerosis, cardiovascular disease, cachexia and anemia, whereas immunosuppression contributes to poor vaccination response and increased incidence and susceptibility to severe infections. In CKD, neutrophils and monocytes display an exaggerated response to stimulation, an increase in TLR expression, and defective phagocytic function. The number of dendritic cells is reduced, as is the expression of MHC class I and class II and costimulatory molecules, leading to an impaired capacity to activate T cells. CKD is also associated with a decreased number of naïve T cells together with an increase in the number of terminally differentiated T cells, which represent a proinflammatory phenotype (7). The number of naïve B cells in CKD patients also decreases due to an increased rate of apoptosis, leading to impaired humoral immunity (8). Collectively, these alterations contribute to compromised innate and adaptive immune responses and chronic-low grade inflammation in CKD patients.

### The Risk of COVID-19 Infection in CKD Patients

Many underlying medical conditions have been linked to increased severity and mortality of COVID-19. Male sex, older age, current smoking habit, obesity, elevated D-dimer level, diabetes, hypertension, chronic obstructive pulmonary disease, malignancies, cardiovascular disease, and CKD have been reported to be risk factors associated with fatal outcomes of COVID-19 (9). CKD patients have increased susceptibility to infections due to an attenuated response of both the innate and adaptive immune systems, consequently leading to worse outcomes of COVID-19 compared with those of patients without CKD. Patients with CKD have an elevated risk of hospitalization, severe disease course and COVID-19-related death (10). In systematic reviews comparing CKD subgroups, a higher risk of COVID-19 mortality was observed with higher stages of CKD (10). In addition, patients with end-stage kidney disease are especially susceptible to SARS-CoV-2 infection and severe COVID-19 (11). CKD has emerged as the most prevalent risk factor for severe COVID-19, preceded only by age (12).

## Impact of Vitamin D on the Immune System and Renin-Angiotensin-Aldosterone System (RAAS)

### Vitamin D and the Immune System

Vitamin D is a fat-soluble essential vitamin that has a substantial role in the modulation of both innate and adaptive immune responses (13). Vitamin D receptors and 1α-hydroxylase are present in several immune cells, including neutrophils, macrophages and dendritic cells, suggesting the effects of vitamin D on the immune system beyond the musculoskeletal system (14). TLR binding enhances the expression of both 1-α-hydroxylase and the vitamin D receptor, leading to the production of AMPs, including cathelicidin and β-defensin 4 (15). Cathelicidins and β-defensins are important AMPs that enhance immune responses by not only the elimination of pathogenic microbes but also the release of chemoattractants, inducing the recruitment of neutrophils, monocytes, and other immune cells to inflammation sites. Vitamin D is also involved in reducing the cytokine storm induced by the innate immune system. Moreover, vitamin D decreases the maturation and antigen-presenting ability of dendritic cells, leading to alterations of the profiles of T helper cells (Th1, Th2, Th9, Th17) and regulatory T cells, leading to overall suppression of the adaptive immune pathway (16). Regarding B cells, vitamin D is involved in inhibition of the generation of both memory and plasma cells, as well as a reduction in immunoglobulin production by inducing apoptosis of immunoglobulin-producing B cells (17).

### Vitamin D and RAAS

Renin is secreted from the kidneys and cleaves angiotensinogen secreted from the liver to the inactive form angiotensin I. Angiotensin I is subsequently cleaved by angiotensin-converting-enzyme (ACE) into active angiotensin II, which binds to two target receptors, angiotensin receptor 1 or 2 (AT1R or AT2R), to exert its effects (18). As a key component of the RAAS, ACE2 cleaves angiotensin I into angiotensin 1–9, which exerts cardioprotective effects by binding to AT2R, and angiotensin II into angiotensin 1–7, which acts on the MAS receptor pathway, counterbalancing the effect of angiotensin (19). Patients with vitamin D deficiency have increased RAAS activity and angiotensin II levels (20, 21). This finding has been confirmed by several studies showing that vitamin D is a negative regulator of the renin gene, leading to reduced renin synthesis independent of angiotensin II (22, 23). Other studies have shown that vitamin D can enhance the expression of ACE2 and inhibit the activation of NF-κB, a transcription factor that regulates multiple aspects of the immune response in infection (19, 24).

## Vitamin D Action in the Pathogenesis of COVID-19 Among CKD Patients

### COVID-19 Pathogenesis

SARS-CoV-2 binds to target cells via ACE2, which is present in the epithelial cells of the lungs, kidneys, intestines, and blood vessels (25). Once SARS-CoV-2 enters target cells, the virus can trigger an innate or adaptive immune response. TLRs expressed on immune cells have the capacity to recognize viruses, leading to the production of interferon (INF) (26). SARS-CoV-2 may dampen antiviral IFN responses by immune evasion, resulting in uncontrolled virus replication, the subsequent infiltration by neutrophils and monocytes/macrophages and the increased release of proinflammatory cytokines. Additionally, the antigen of SARS-CoV-2 is presented by MHC and then recognized by cytotoxic T lymphocytes (26). Activation of specific lymphocyte T helper cells (Th1/Th17) may also lead to aggravated inflammatory responses. Viral antigens can also be recognized by B cells/plasma cells, which are activated to produce specific antibodies to neutralize SARS-CoV-2 and provide systemic immunity in different organs. Collectively, the overall immune response involves the sustained production of proinflammatory cytokines such as TNF-α, IL-6, and IFN-α/-γ, resulting in “cytokine storm,” accompanied by a reduction in anti-inflammatory cytokine levels (27). Overwhelming viral replication together with cytokine storm induces considerable damage to bodily tissues with endothelial injury and thrombotic microangiopathy, leading to ARDS, respiratory failure, sepsis, heart failure and thrombotic complications, which have been reported as the most common causes of death.

ACE2 not only serves as the point of cellular entry for SARS-CoV-2 but also might be involved in COVID-19 pathogenesis. Upon infection, SARS-CoV-2 downregulates ACE2 expression, leading to decreased downstream conversion of angiotensin II to angiotensin 1–7 and angiotensin 1–9 (28). This imbalance in RAAS regulation increases angiotensin II concentrations and upregulates the AT1R pathway, resulting in excessive production of proinflammatory cytokines and chemokines and the subsequent initiation of cytokine storm (29).

### Vitamin D Deficiency in CKD Patients

Vitamin D is produced predominantly in skin exposed to ultraviolet B radiation, and only 10% is absorbed from the diet. The kidneys play an important role in the metabolism of vitamin D in the body. It is hydroxylated first to 25-hydroxyvitamin D in the liver and then to its active form, 1,25-dihydroxyvitamin D, in the kidney by the enzyme 1α-hydroxylase. Cholecalciferol/ergocalciferol synthesis in the skin is reduced in patients with uraemia due to skin discolouration or hyperpigmentation and reduced exposure to sunlight. Additionally, dietary restriction and protein-energy wasting may also lead to decreased vitamin D intake. CKD patients also have reduced 25-hydroxyvitamin D levels because of a lack of its precursor, urinary loss in nephrosis, and sequestration in the body fat compartment due to a higher percentage of obese patients in the CKD population (30). Furthermore, the 1,25-dihydroxyvitamin D level is reduced in CKD patients because hyperphosphataemia, metabolic acidosis, and elevated levels of fibroblast growth factor 23 can suppress 1α-hydroxylase activity. Finally, a decrease in the number of functioning renal tubules results in lower 1,25-dihydroxyvitamin D production in patients with advanced CKD (31).

### Vitamin D and Respiratory Tract Infections

Several studies have shown that vitamin D deficiency is associated with higher susceptibility to serious viral respiratory tract infections (13). The potential benefits of vitamin D on immune modulation are due to crosstalk between vitamin D metabolism and signaling and both innate and adaptive immunity. Recent evidence has shown that vitamin D promotes immune sensing of respiratory viral infections, including influenza A and B, parainfluenza 1 and 2, and respiratory syncytial virus (32). A systematic review of clinical studies showed evidence linking low vitamin D levels and increased risk of both upper and lower respiratory tract infections, suggesting a role of vitamin D in the prevention of respiratory tract infections (33). Additionally, vitamin D deficiency can amplify the risk of non-COVID respiratory tract infections (34–37) and has been associated with worse outcomes (38). However, randomized controlled trials addressing the hypothesis that vitamin D could reduce the risk of respiratory tract infections have obtained conflicting results, possibly due to the different dosing regimens of vitamin D administered and serum levels of vitamin D at baseline (33).

### Vitamin D and COVID-19

Vitamin D may provide protection against COVID-19 infection and modulate the severity of its outcome (39, 40). Previous studies have demonstrated that SARS-CoV-1 inhibits type 1 IFN receptors, which can attenuate host innate immune responses (41). Calcitriol can bind to the vitamin D receptor to enhance the type 1 IFN response and improve the innate immune response (42). The production of AMPs in airway epithelial cells is induced by vitamin D, making infection with SARS-CoV-2 and the development of severe COVID-19 less likely (43). Vitamin D might help to reduce the inflammatory response to infection with SARS-CoV-2 (13). As a binding protein for viral entry into host cells, the expression of ACE2 decreased during SARS-CoV-2 infection. Vitamin D can modulate the RAAS pathway and increase ACE2 expression, thus potentially protecting against severe lung injury (44). In addition, vitamin D acts as a negative acute phase reactant in most acute and chronic inflammatory diseases (45). Vitamin D can acidify endolysosomes and enhance autophagy, and thus, it might promote SARS-CoV-2 degradation (46, 47). Notably, calcium signaling plays important roles in virus entry and gene expression, and the alteration of calcium homeostasis in host cells can benefit virus lifecycles (48). Several studies have reported that hypocalcaemia is commonly observed among COVID-19 patients with severe disease (49, 50). In the setting of chronic vitamin D deficiency, the presence of acute hypocalcaemia in COVID-19 contributes to a unique osteometabolic phenotype (51). Altogether, the aforementioned findings have led to the hypothesis that vitamin D deficiency may increase the risk of COVID-19 infection and predispose patients to worse outcomes.

## Effect of Vitamin D Supplementation on COVID-19

### Vitamin D Levels

Several observational studies have assessed the correlation between vitamin D levels and outcome of COVID-19. Evidence has suggested that vitamin D deficiency is associated with susceptibility to COVID-19 infection (52–55). Most studies suggested that low vitamin D levels among COVID-19 patients increase the probability of hospitalization, severity of disease, and risk of mortality (56–61). In contrast, other studies did not find any correlation between vitamin D and COVID-19 (62–64). These findings suggest that vitamin D supplementation may be a potential adjunct treatment to reduce the risk for SARS-CoV-2 infection and COVID-19. Of note, several factors may influence the assessment of vitamin D levels, such as acute critical illness. 25-hydroxyvitamin D levels are usually lower in patients hospitalized with COVID-19, and blood samples collected during critical illness may be prone to spurious correlations. Additionally, studies using retrospectively obtained vitamin D levels before hospitalization may have bias, as these patients have increased interaction with healthcare providers (65).

### Clinical Studies in the General Population

Currently, the number of clinical trials investigating the effects of vitamin D supplementation on recovery from COVID-19 infection is limited but increasing. The results of observational studies assessing the role of vitamin D supplementation in COVID-19 patients remain a matter of debate, wherein there is a higher risk of mortality among patients with calcifediol supplementation (66, 67), a lack of association between vitamin D supplementation and clinical outcomes (64), and improved clinical outcomes among patients supplemented with vitamin D (68–71). Some clinical trials also showed improved survival of COVID-19 patients who were supplemented with vitamin D (72, 73). To date, only 11 randomized controlled trials assessing the effect of therapeutic vitamin D supplementation on COVID-19 patients have been published; however, the role of vitamin D in disease severity remains controversial (42, 74–83). Most of these trials showed beneficial effects on a reduction in inflammatory markers, improvements in immune function, SARS-CoV-2 viral clearance, recovery of symptoms, and reductions in the severity of the disease and in-hospital mortality (42, 74, 75, 77–80). Nevertheless, other studies demonstrated contradictory data that vitamin D supplementation did not improve inflammatory markers or reduce the length of hospital stay, admission to the intensive care unit or mortality (76, 81–83). Most of these trials were limited by variable vitamin D absorption and sun exposure because of different ethnic groups, seasons, and geographic latitudes in the study population. Additionally, the supplemented dosage of vitamin D varies in these studies, as do the assays used for laboratory measurement of vitamin D levels. Regarding systematic reviews and meta-analyses, a previous review that included three studies did not show the effectiveness of vitamin D supplementation on mortality in COVID-19 patients (84). However, a recent meta-analysis found that vitamin D supplementation may be beneficial for clinical outcomes of COVID-19, especially when treatment begins following the diagnosis of COVID-19 (85).

### Clinical Studies in CKD Patients

Currently, clinical studies evaluating the effects of vitamin D supplementation on COVID-19 in patients with CKD are extremely limited because this specific population is often excluded from ongoing clinical trials. To our knowledge, only four observational studies have been published (Table 1). In a case series of non-vaccinated haemodialysis patients with SARS-CoV-2 infection, chronic active vitamin D treatment was associated with a reduced risk of severe COVID-19 (88). Another retrospective observational study conducted on haemodialysis patients with COVID-19 showed that those who received paricalcitol, calcimimetics or the combination of both exhibited improved survival (87). In a large population-based cohort study, calcitriol supplementation showed a beneficial effect on a reduction in disease severity and mortality of COVID-19, particularly in patients with advanced CKD (86). Last, a recent retrospective cohort study showed that there was a trend toward a reduction in COVID-19 mortality among patients with advanced CKD supplemented with calcifediol (89). Among these studies, three showed significant beneficial effects of calcitriol or vitamin D receptor activator analog (paricalcitol) while one study demonstrated the potential role of calcifediol. In CKD, hypocalcaemia and 1,25-dihydroxyvitamin D deficiency contribute to the development of secondary hyperparathyroidism and consequently mineral and bone disorders that are frequently treated with active vitamin D (90). However, the use of active vitamin D decreases the 1α-hydroxylase and 25-hydroxylase activity and increases the 24-hydroxylase activity, leading to increased production and decreased degradation of 25-hydroxyvitamin D and 1,25-dihydroxyvitamin D (91). Because 1-hydroxylase and ACE2 are both expressed in renal proximal tubular cells, SARS-CoV-2 infection may decrease generation of 1,25-dihydroxyvitamin D which cannot be replenished with nutritional vitamin D supplementation (cholecalciferol or ergocalciferol) (92). Additionally, calcitriol has a higher affinity for the vitamin D receptor and greater potency to exert its biological activity, including immune modulation and RAAS regulation. Collectively, supplementing active vitamin D or its analogs restores the physiologic levels of the active vitamin D hormone and may be more effective than nutritional vitamin D in CKD patients with COVID-19. Further randomized, interventional trials are warranted in patients with CKD to clarify which vitamin D supplementation would be beneficial for recovery from COVID-19.

**Table 1:** Clinical studies evaluating vitamin D effects on COVID-19 in CKD patients.

**Reference**	**Design, country**	**Participants**	**Mean age, sex**	**Conclusions**
Oristrell et al. (86)	Observational, Spain	Overall cohort: 6252 subjects on calcitriol and 12,504 matched controls Subgroup analysis: 2296 stage 4 or 5 CKD subjects on calcitriol and 3407 matched controls	70.2 ± 15.6 years old v.s. 70.7 ± 14.7 years old, female 57.5 v.s. 57.5%	Calcitriol use reduce risk of SARS-CoV2 infection, severe COVID-19, and mortality in stage 4 or 5 CKD
Arenas Jimenez et al. (87)	Observational, Spain	288 HD patients with COVID-19 Vitamin D treatment includes calcifediol, calcitriol, and paricalcitol. Calcimimetic includes cinacalcet and etelcalcetide	72.4 ± 12.6 years old, female 29.2%	Paricalcitol, calcimimetics or combination reduce mortality rate
Tylicki et al. (88)	Case series, Poland	85 nonvaccinated HD patients with COVID-19	69.74 ± 13.19 years old, female 47.06%	Chronic active vitamin D treatment reduce the risk of severe pneumonia
Oristrell et al. (89)	Observational, Spain	Overall cohort: 134,703 patients on calcifediol and 269,406 matched control Subgroup analysis: 130,323 stage 1–3 CKD subjects on calcifediol and 263,873 matched controls; 4380 stage 4 or 5 CKD subjects on calcifediol and 5533 matched controls	68.8 ± 14.9 years old v.s. 68.8 ± 15.1 years old, female 78.1 v.s. 77.9%	Calcifediol supplementation reduce risk of SARS-CoV2 infection and severe COVID-19 and a trend toward a reduction in mortality in stage 4 or 5 CKD

*CKD, chronic kidney disease; COVID-19, coronavirus disease 2019; HD, haemodialysis*.

## Conclusions

As a respiratory infection, COVID-19 may affect multiple organ systems and contribute to infection-related tissue damage. Vitamin D is an immunomodulator hormone and has been shown to have protective effects against respiratory infections. Accumulating evidence has shown that vitamin D is associated with improved clinical outcomes in terms of disease severity and/or mortality during the COVID-19 pandemic, although current data from the CKD population are still limited. Patients with CKD are characterized by the coexistence of immune activation and immune deficiency and are at increased risk for vitamin D deficiency. Supplementing vitamin D in this vulnerable population may be a critical form of support and is helpful for the recovery from COVID-19. Current observational data provide avenues for further research, including randomized controlled trials, to determine the effect of vitamin D supplementation on COVID-19 among CKD patients.

## Author Contributions

W-FC wrote the manuscript. P-JH conceived and organized the structure of the review. J-SC contributed to the critical revision of the paper. All authors approved the final version of the manuscript and ensured the accuracy and integrity of the work.

## Funding

This study was supported in part by a grant from the Armed Forces Taoyuan General Hospital (TYAFGH-D-111028).

## Conflict of Interest

The authors declare that the research was conducted in the absence of any commercial or financial relationships that could be construed as a potential conflict of interest.

## Publisher's Note

All claims expressed in this article are solely those of the authors and do not necessarily represent those of their affiliated organizations, or those of the publisher, the editors and the reviewers. Any product that may be evaluated in this article, or claim that may be made by its manufacturer, is not guaranteed or endorsed by the publisher.
